# Going beyond the technology: Considerations in translating electronic case report forms

**DOI:** 10.1017/cts.2025.10078

**Published:** 2025-06-18

**Authors:** Amy E. Krefman, Luke V. Rasmussen, Crystal Santillanes, Mary Beth Tull, Patricia Bustamante, Alan Kuang, Hongyan Ning, Yaojie Wang, Barbara M. Gonzalez, Eugenia I. Manrique, Lauren B. Bonner, Jody D. Ciolino

**Affiliations:** 1 Preventive Medicine, Northwestern University Feinberg School of Medicine, Chicago, IL, USA; 2 Miller School of Medicine, University of Miami, Miami, FL, USA

**Keywords:** Translating, diversity, equity, inclusion, data collection, workflow

## Abstract

Through a series of example research studies, we illustrate processes in translating case report forms to increase language diversity in study populations while simultaneously highlighting implications for data collection and analyses. The Northwestern University Data Analysis and Coordinating Center manages the translation of participant-facing study documents into languages other than English through a process that has been refined over several years, adjusting for changes in technical capabilities in electronic case report forms. This approach to manage, examine for context, and implement certified case report form translations offers an efficient workflow to streamline data capture in multiple languages.

## Introduction

When conducting clinical research, it is important to enroll participants belonging to under-represented populations to ensure a representative sample and increase generalizability [1].The translation of all participant-facing materials, including case report forms (CRFs), is critical to mitigate recruitment barriers for participants who may not speak English and thus retain a representative research study population. Depending on review board requirements, a certified translation service may be used [2] to translate CRFs, but ensuring usability and flexibility in function for the study participants requires an investment of time, money, and thoughtful consideration.

The Food and Drug Administration (FDA) does not have nuanced guidance on requirements for translating study materials such as informed consent documents [2]. The FDA requires more than translation alone when a patient-reported outcome serves as an endpoint; they require validation and cultural adaptation [3]. However, many studies not regulated by the FDA enroll participants across multiple languages. Despite budgeting for translations, some research teams are not able to validate or evaluate questionnaires through a rigorous cultural adaptation process. Participants tend to prefer questionnaires that undergo a cultural adaptation process over those that undergo forward-back translation (i.e., one translator translates the document into the new language, and a second translator translates it back to the original language, and the two versions are compared) [4]. Cultural adaptation processes are especially helpful for identifying intangible cultural heritage terms, which are “unique expressions of cultural knowledge and practices that are deeply rooted in a particular region or community” [5]. However, cultural adaptation is expensive, and one recognized barrier to enrolling patients from minority populations is lack of budget for translating study materials, especially for investigator-initiated studies [6].

Additionally, different translation approaches affect the “readability, comprehension, and user preferences” of participant-facing materials [7]. These translation approaches include literal, functionalist, and equivalence-based approaches, with a preference for the functionalist approach—where a translator makes decisions based on instructions meant to better suit the needs of the target audience [7]. However, investigator-initiated studies in academia often lack personnel and resources to vet and compare translation companies on these approaches. Further, translation companies may not have the necessary contextual understanding for a clinical trial or observational study to implement a functionalist approach, and some vendors’ policies hold the requestor accountable for the translation context.

Finally, implementation of the translated study materials can be difficult. Some electronic data capture (EDC) systems can accommodate the same case report forms in multiple languages, but many do not. It is important to consider the end user experience when planning a study’s translations, which in our experience requires careful attention to interface details and functionality (e.g., the systems navigation buttons such as “next” or “submit” may also require translation).

## Objective

Leveraging REDCap [8,9] and a highly collaborative multi-disciplinary team, we developed a multi-step process to manage, implement, and certify language translations to increase representation from study participants whose primary language is not English. This process increases data integrity by using written translations that captured data in a study participant’s language of choice and uses the same variable names across languages to facilitate data analysis.

## Materials and methods

The Northwestern University Data Analysis and Coordinating Center (NUDACC) serves as the data coordinating center for multiple prospective clinical studies, managing all aspects of study design, database development, data collection, data quality control, regulatory reporting, and statistical analyses. Given these studies enroll participants across geographically diverse regions throughout the United States, it is imperative to reduce barriers to enrollment for individuals whose native language may not be English. To ensure representative study samples, NUDACC manages the translation of study documents into languages other than English. Depending on the needs of the study, translated documents may include Informed Consent Forms, recruitment materials (e.g., brochures, advertisements, websites), and participant-facing CRFs. As part of ongoing internal process improvement efforts, NUDACC conducts periodic retrospective assessments of study operations where members of the team engage in open dialog about what went well, what could be improved, and adapt workflow for ongoing and future studies. This involves discussions amongst a collaborative study team with statistical, informatics, clinical research, regulatory, and project management expertise.

NUDACC’s team members have been supporting studies requiring CRF translations for over a decade, and here we touch on several NUDACC-specific and NUDACC-affiliated studies that serve to illustrate how CRF translation processes have been implemented: QUARTET [10,11], Mothers and Babies [12], GO MOMs [13], and most recently the Liver Cirrhosis Network (LCN) [14]. These studies have provided opportunities to implement and refine our strategies for managing CRF language translations into a process that accounts for data structure and the intricacies of language in the clinical research context. Here we describe key study elements, system functionalities, and decisions that have helped to inform our proposed workflow, the NUDACC translation workflow (NTW).

### Mode of delivery

**Figure 1.**
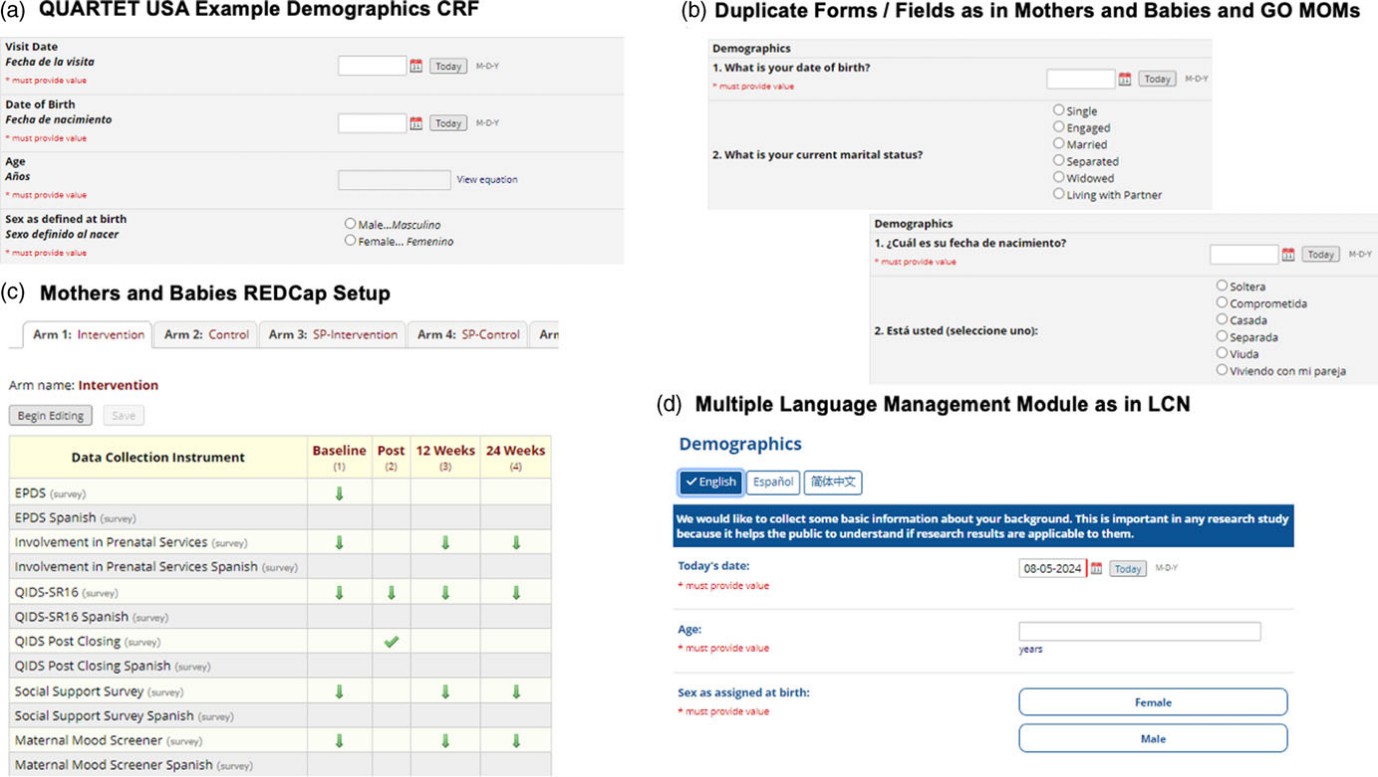
Overview of REDCap Setup to Accommodate eCRF Translations across Multiple Studies (a) In this example demographics form from the QUARTET USA study, both English and Spanish labels for questions and responses are included for any one field. Upon export (the back end), the programmer would need to only use one set of fields for data merging, restructuring and analyses; however, with certain fields and responses, this setup may be visually overwhelming for the data enterer or study participant taking the survey. (b) The Mothers and Babies study duplicated all surveys (participant-facing eCRFs) and used the “arm” feature in REDCap to delineate between participants taking the surveys in English (depicted here) and those that are taking the surveys in Spanish (in Arms 3 and 4, with the corresponding Spanish forms linked to the appropriate events. (c) In both the Mothers and Babies and the GO MOMs study examples this requires duplicate sets of forms and thus duplicate sets of fields, resulting in more programing and merging on the back end prior to analyses. (d) The Multiple Language Management Module allows the data enterer/study participant to toggle to their preferred language in real-time. Upon export, the programmer will only have one set of fields to use. QUARTET = Quadruple Ultra-low-dose Treatment for Hypertension; USA = United States of America; CRF = case report form; eCRFs = electronic case report forms; GO MOMs = Glycemic Observation and Metabolic Outcomes in Mothers and Offspring study; REDCap = Research Electronic Data Capture; LCN = Liver Cirrhosis Network.

Varying study needs and platform capabilities at the time of database development may require different approaches, for example: (1) creating duplicate electronic CRFs (eCRFs) within the same database for each language, (2) creating separate databases for each language, (3) modifying field labels to include translated text, or (4) using REDCap’s [8,9] Multi-Language Management (MLM) module to translate eCRFs within the same project (Figure 1). These strategies have their own strengths and limitations for database creation, data collection, monitoring, and eventual analysis and are described further in Table 1 and in Supplemental Methods. Regardless of the technical implementation, all methods require additional effort to ensure the translated material conveys the intended context while brokering cultural sensitivity. While the process we continue to describe here is centered on REDCap’s MLM, there are other EDC systems with similar translation capabilities such as ClinInfo’s ePro [15], OpenEDC [16] and OpenClinica [17].

**Table 1.** Description of example studies and multiple language display set up in REDCap

Example Study (clinicaltrials.gov identifier)	Study Design	Languages	REDCap Setup and Functionality**	Pros	Cons
**QUARTET USA** (NCT03640312) Start Year: 2019	Double-blind Randomized Controlled Trial (RCT)	English Spanish	English and Spanish labels for questions and responses on the same CRF	No need to merge multiple fields on export Less room for programing errors on export, especially for programmers unfamiliar with second language	Visually overwhelming for participant Likely only possible with 2 total languages Requires manual programing of field labels and code list options within REDCap Built-in buttons and commands cannot be translated (e.g., “next page,” “submit”)
**Mothers and Babies** (NCT02979444) Start Year: 2016	Cluster-randomized trial	English Spanish	Spanish versions of assessments in a separate “arm” in REDCap; separate Spanish forms	Easier for coordination and data entry team to determine language and set of forms to send to participants Translation of CRFs is “cleaner” and more efficient	Requires substantial programing and merging of fields on export Opportunity for programing errors – both on merging data for programmers and in creating translated forms for those building database Built-in buttons and commands cannot be translated (e.g., “next page,” “submit”)
**GO MOMs** (NCT04860336) Start Year: 2021	Prospective observational study	English Spanish	Separate Spanish forms and data are backfilled into English forms via programing script nightly if filled out in Spanish	Same pros as “Mothers and Babies” Study Backfilled Spanish to English makes monitoring possible for non-Spanish-speaking monitors	Same cons as “Mothers and Babies” Study except, because each language has a separate CRF, it is not visually overwhelming to the participant. Code to backfill Spanish to English takes time to generate and troubleshoot – added opportunity for programing errors
**Liver Cirrhosis Network** (NCT05740358; NCT05832229) Start Year: 2022; 2023	Prospective observational study Double-blind RCT	English Spanish Simplified Chinese	Multiple Language Management module	Easier for coordination team or participant to select the language they prefer for response No need to merge multiple fields on export Less room for programing errors on export, especially for programmers unfamiliar with second language Built-in buttons and commands can be translated and added into end user interface (e.g., “next page,” “submit”) Can efficiently handle >2 languages	Requires substantial upfront programing and testing during database development

QUARTET = Quadruple Ultra-low-dose Treatment for Hypertension; USA = United States of America; CRF = case report form; GO MOMs = Glycemic Observation and Metabolic Outcomes in Mothers and Offspring study; REDCap = Research Electronic Data Capture; RCT, Randomized Controlled Trial.

**Refer to Figure 1 for visual representation.

## Results

### NUDACC translation workflow

**Figure 2.**
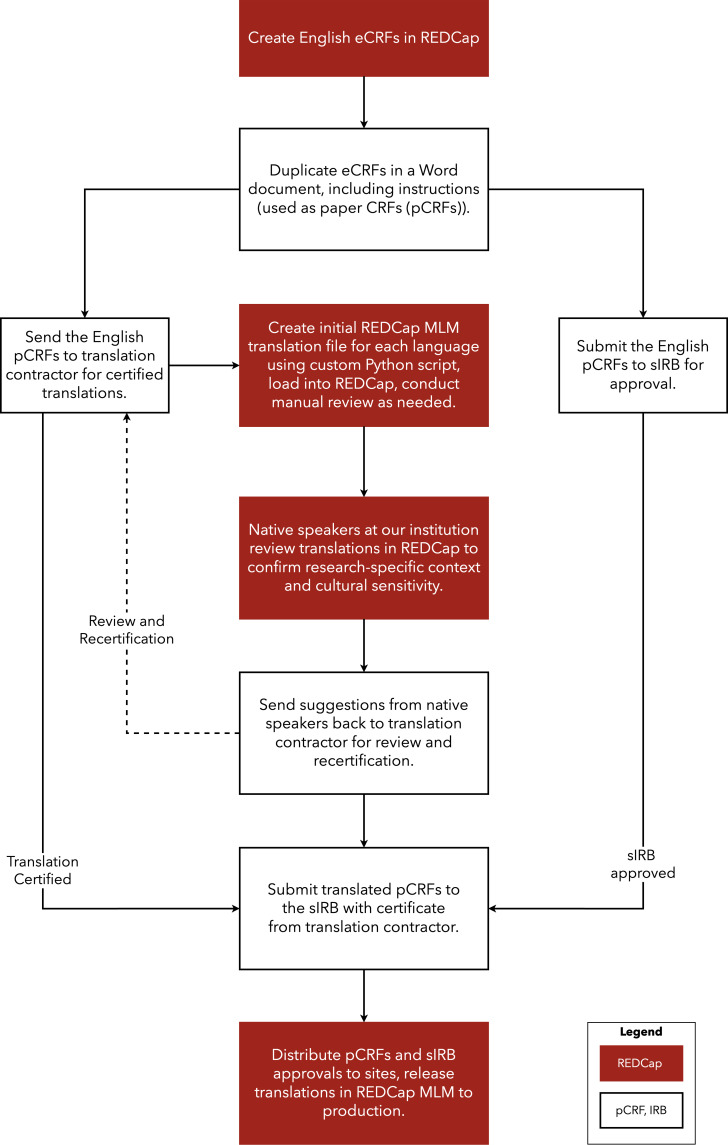
Flow chart describing the NUDACC translation workflow process. ECRFs = electronic case report forms; pCRFs = paper case report forms; REDCap = Research Electronic Data Capture; MLM = Multi-Language Management module; Sirb = single Institutional Review Board.

Leveraging the team’s experience with translation of study materials as described above, we developed a workflow that provides an efficient and effective process for translating study documents; implementing translations into an electronic database; maintaining alignment between paper CRFs (pCRFs) and eCRFs; ensuring readability and comprehension in multiple languages; and facilitating data monitoring, reporting, and analysis (Figure 2).

The NUDACC translation workflow (NTW) begins by creating an eCRF in the primary language (in our case, English) in REDCap, for any participant-facing forms. Once the eCRF is created, it is then duplicated in a word processing document to act as the pCRF, rather than printed directly from REDCap, to allow for explicit instructions and branching logic. We note non-technical challenges in this process. For instance, instructions may need to be modified to account for inherent differences between paper and electronic form instructions (e.g., “click” vs “circle,” Supplemental Figure 1). This primary language pCRF is simultaneously submitted to the Institutional Review Board (IRB) and a translation contractor for certified translations into the target language(s). We also include a separate document of “supplemental translations” that may include eCRF-specific instructions such as page actions (e.g., “close,” “cancel,” “confirm”) and site navigation (e.g., “next page,” “previous page”) that the end user may see.

To utilize the REDCap MLM, described in detail in Supplemental Methods, we first create an initial MLM translation file for each language using a custom Python script shown in Supplemental Figure 2. We load each translation into REDCap, conducting a preliminary manual review and modification of any errors as needed. This script allows programmers who are unfamiliar with the language or alphabet used to successfully implement MLM, as long as the format and structure of the translation source documents remain consistent. The REDCap community also provides a shared library of translated phrases for the user interface—such as elements used in surveys or the survey submit buttons—and their certificate of translation [18].

Once the translation is in place in REDCap, we asked native speakers (for LCN, this included both Spanish and Mandarin speakers) to review the translations using the MLM and/or pCRFs as our language contractor may not always have the research context to choose the best word or phrase. For instance, for the LCN study, we included a questionnaire about “food security,” which refers to how easily one feels they can access the food they need. Translators initially used a word that referred to the security of food quality (“is it safe to eat”), rather than the security of food access. Without the added step of native speaker review for the certified translations, the research-specific context would have been missed. Any suggestions are sent back to the translation contractor for review and recertification. Once the translations are finalized and the English versions are IRB-approved, we submit the translated pCRFs to the IRB with the certificate from the translation contractor. Finally, we distribute the pCRFs and sIRB approvals to sites, and release translations in REDCap MLM to production.

Once a study is up and running with translated participant materials, it is important to keep the translated participant materials synchronized with current participant materials. Any multi-centered trial will have a series of modifications, including adding or editing participant materials, throughout its lifecycle. During modifications, small changes may occur such as adding or removing text to clarify instructions. All changes undergo the entire process of (1) certified document translation, (2) native review, (3) recertification of translation with proposed native review changes; and (4) submission to IRB.

### Impact

Using NTW, LCN has translated 15 CRFs into two languages, and are enrolling participants who complete surveys in English, Spanish, and Simplified Chinese. During development, native speakers were able to identify multiple errors in both the original translation and REDCap programing, which highlights the need for this step in NTW. Contrary to other options presented in Table 1, study statisticians can quickly and easily query data completion and quality by language to spot any trends or issues in translation as the study progresses, and the burden of data restructuring by language is eliminated. Recruitment is ongoing and participants in all three languages have been enrolled.

## Discussion

REDCap and other EDC systems support multiple ways to implement translated eCRFs. Here we describe a process that includes several considerations beyond the technical implementation that are critical to ensure similar engagement and comprehension from non-English speaking participants as compared to native English speakers. NTW requires additional time and budget, native-speaking personnel, and regulatory expertise as well as complex document management are crucial. Implementing NTW requires the largest up-front investment with the first set of translations; however, the workload diminishes greatly once the materials are finalized.

Our translation process navigates some of the issues posed by other research teams such as Colina et al., including readability, comprehension, and data validity [7,19]. For studies unable to undertake full validation, cultural adaptation, and a functionalist approach, we proposed a straightforward alternative that incorporates review by native speakers who can provide both cultural heritage and the research knowledge to avoid the common mistakes of translating, not translating, or providing out of context study materials.

One challenge we encountered was accommodating regional dialects – for example, several studies include Spanish-speaking participants living in Florida and Southern California who speak different dialects. This required occasional discussion amongst the translation service and our native speakers to agree on appropriate phrasing. We also acknowledge the added care needed for translating validated questionnaires; in this case close collaboration with the study investigators identified existing validations and copyright issues. Additionally, appropriate reading level of translated text can be easily overlooked when translating study materials if not explicitly indicated.

We acknowledge this work has several limitations. NTW does not follow a cultural adaptation process, does not produce validated research instruments (though validated instruments can be incorporated into the MLM in REDCap, benefitting from the same data structure advantages), and we did not incorporate a focus group or review by our participant population. The pool of native speakers reviewing the translation in LCN were not representative of all Spanish-speaking regions, for example Spain, which often diverts from other dialects in both word choice and pronunciation. As data collection for LCN is ongoing, we were not able to directly compare data quality between NTW and previous methods. Last, while we believe NTW may be more cost-effective than other alternatives, future work should include a formal cost comparison study.

## Conclusion

The multi-step translation process for CRFs proposed here offers an adaptable approach that can be implemented using a variety of EDC systems. NTW allows for increased representation in clinical study populations, improved understanding of questions and responses provided in a participant’s primary language, a streamlined alternative for investigator-initiated studies, and more straightforward data processing.

## Supporting information

10.1017/cts.2025.10078.sm001 Krefman et al. supplementary material Krefman et al. supplementary material

## Data Availability

No new data were generated or analyzed in support of this research.

## Supplementary material

The supplementary material for this article can be found at https://doi.org/10.1017/cts.2025.10078.
