# Supplementary material for: Going beyond the technology: Considerations in translating electronic case report forms
Source: J Clin Transl Sci. 2025 Jun 18;9(1):e150. doi: 10.1017/cts.2025.10078 (PMC12930200; doi:10.1017/cts.2025.10078)
Supplement: Krefman et al. supplementary material [file S2059866125100782sup001.docx]

**Supplemental Figure 1.**

**
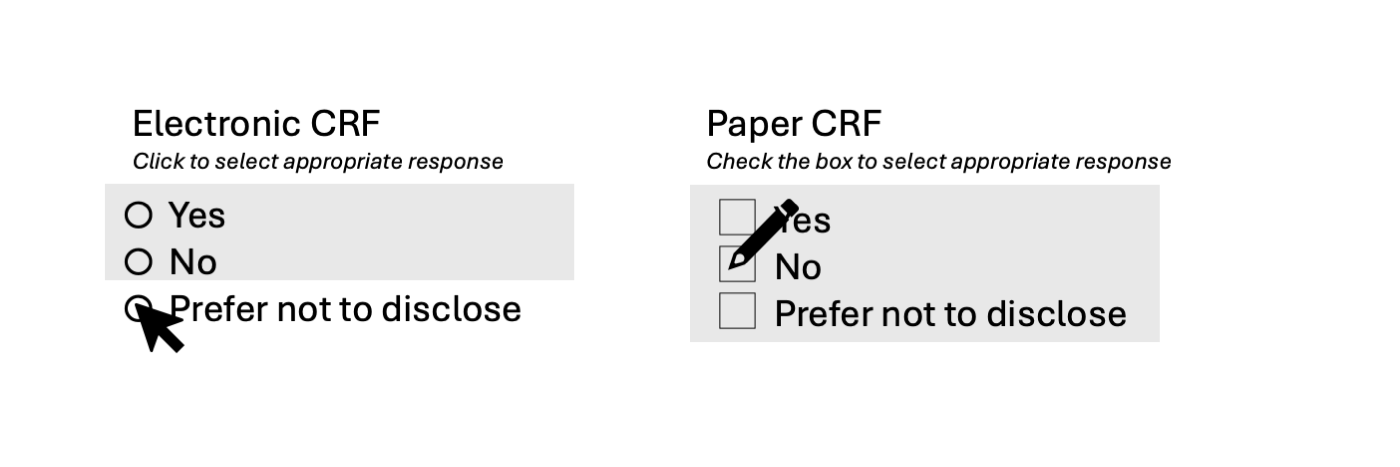
**

**Supplemental Figure 2.**

**
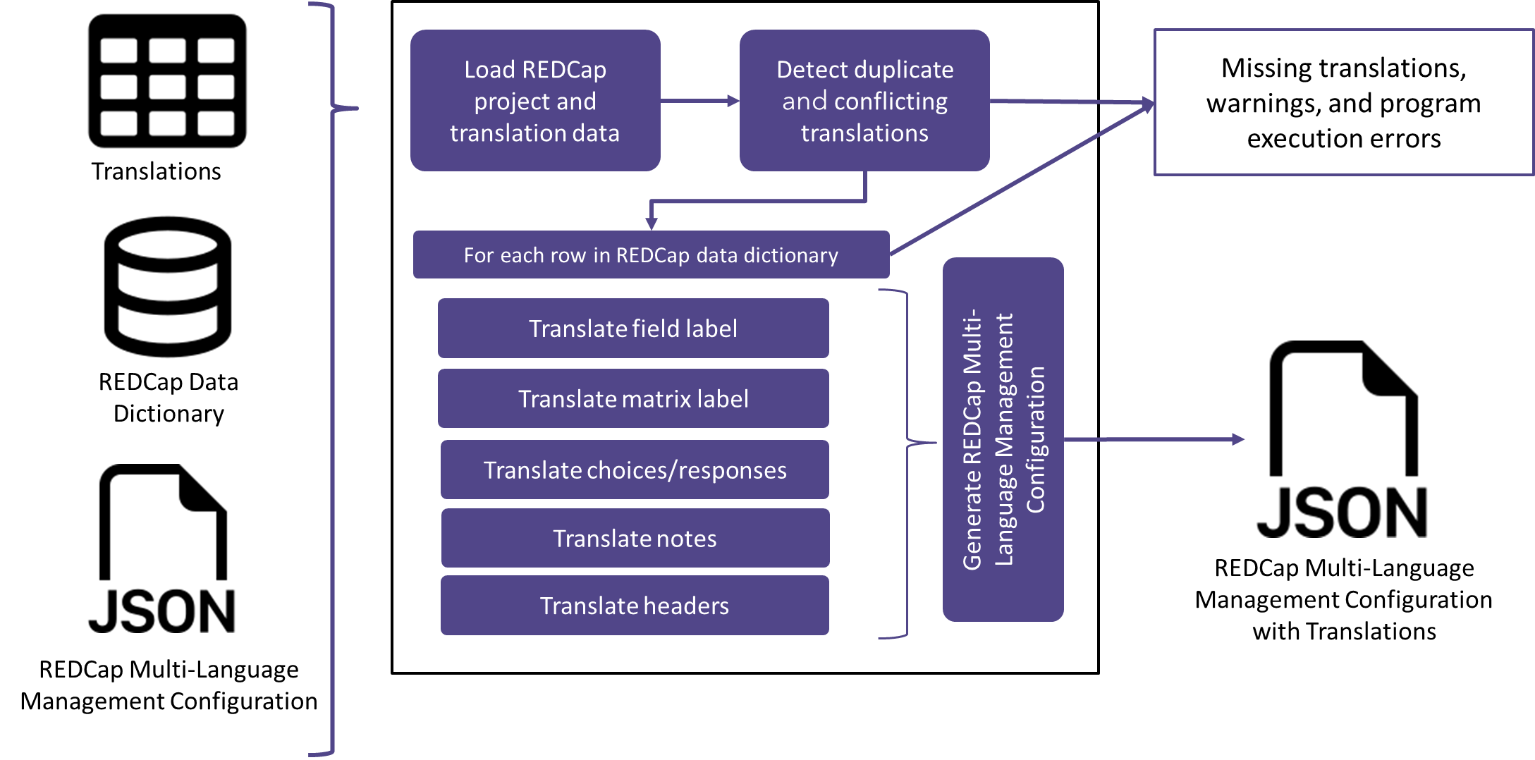
**

**Supplemental Methods.**

Over time, we have refined our strategies for managing eCRF translations. Here we describe the processes we utilized in REDCap, using the modules and capabilities that were available at the time of database development. Different structures may work better for different study schedules, populations, and data types.

QUARTET USA: In this study’s forms set, the team decided to include both English and Spanish labels for questions and responses on same survey or CRF. This required upfront programming on the data collection tools themselves within REDCap, but it made merging and restructuring on the back end much simpler than perhaps some of the other options. Refer to Figure 1a for an example of a small number of fields from the Demographics survey for this study. The drawbacks of this setup, however, include: (1) potential visual overwhelm for the data enterer or study participant, especially for longer questions or responses; (2) this method would likely only be feasible for 2 total languages as adding another language would only magnify the issue of visual clutter; (3) the person programming the field labels and options in REDCap must manually take the translated elements and insert them into the data dictionary one-at-a-time, resulting in large upfront programming costs; (4) since the REDCap instance is in English, the built-in survey options cannot be translated for the participant.

Mothers and Babies: In this study’s database, the team made use of the “arm” feature in REDCap. Participants receiving intervention had a slightly different forms set and schedule than participants receiving control. We reserved Arms 1 and 2 for these participants, respectively, linking forms to the study time points differently per study arm. The team decided to create a separate set of forms in Spanish for each survey, and there were about 20 surveys total that were initially programmed in English. Thus, those programming the database in REDCap created about 20 duplicate surveys in Spanish and reserved those Spanish versions of assessments for intervention and control participants for REDCap Arms 3 and 4, respectively. Refer to Figure 1b and 1c. The primary drawback of this setup lies in the number of forms and fields and the heavy programming requirements on the back end upon data export. Not only do fields require merging, but using different arms in REDCap will create separate sets of rows per participant in each intervention group/language that will require merging.

GO MOMs: In GO MOMs, a combination of English/Spanish labels for questions and responses on the same CRF and a separate Spanish CRFs of the English equivalent are utilized. In the latter method, scheduled scripts are performed daily early in the morning to backfill the English CRFs with the responses from the Spanish CRFs. The English/Spanish CRFs pairs have the same variable name, with “_sp” appended to the back of all the Spanish CRFs. The variables are then matched to their English counterparts and backfilled with the data entered in the Spanish, as both the English and Spanish variables are coded the same, no additional step in modifying the data is required before backfilling.

Liver Cirrhosis Network (LCN): REDCap Multi-Language Management

This module within REDCap allows for translation of all participant-facing content including: form names, field labels, choice/option labels, user interface elements (e.g., “Submit”, “Next”, “Save”), data-entry validation pop-up messages, emails, alert messages, survey settings, and survey invitations. While MLM does not translate content, it provides the framework and functionality for incorporating translated material into the database. Once enabled, study participants can toggle between languages allowing them to respond to questionnaires in their preferred language (Figure 1d). On the back end however, questions answered in different languages correspond to the same variable, using the preferred variable naming mechanism and coding of the study team. This ultimately avoids the need for restructuring data captured in different languages. For example, data captured on language-specific eCRFs (as in Figure 1b and 1c) will require multiple variables with unique variable names for each language. Reporting and analysis collapsed over languages will involve merging language-specific variables into a single column.
